# Exploring how cognitive-behavioral physical activity links ruminative thinking and mental wellbeing in sports high school adolescents

**DOI:** 10.3389/fpsyg.2025.1665882

**Published:** 2025-10-21

**Authors:** Mehmet Derelioğlu, Mustafa Vural, Erkan Çimen, Ünal Saki, Yusuf Yağız Saraçoğlu, Baykal Karataş, Ajlan Saç, Emre Yamaner, Medera Halmatov, Coşkun Yılmaz, Mehmet Öztaş, Gökhan Arıkan, Levent Ceylan

**Affiliations:** ^1^Department of Exercise and Sport Science, Faculty of Sports Sciences, Avrasya University, Trabzon, Türkiye; ^2^Department of Sport Management, Faculty of Sports Sciences, Agri Ibrahim Cecen University, Agri, Türkiye; ^3^Department of Physical Education and Sport, Faculty of Sports Sciences, Suleyman Demirel University, Isparta, Türkiye; ^4^Department of Physical Education and Sport, Faculty of Sports Sciences, Agri Ibrahim Cecen University, Agri, Türkiye; ^5^Department of Coaching Education, Faculty of Sports Sciences, Agri Ibrahim Cecen University, Agri, Türkiye; ^6^Department of Coaching Education, Faculty of Sport Sciences, Trakya University, Edirne, Türkiye; ^7^Sungurlu Vocational College, Hitit University, Corum, Türkiye; ^8^Department of Child Development, Faculty of Health Sciences, Bilecik Seyh Edebali University, Bilecik, Türkiye; ^9^Department of Management and Organization, Kelkit Aydin Dogan Vocational College, Gumushane University, Gumushane, Türkiye; ^10^Department of Physical Education and Sport, Faculty of Sports Sciences, Tokat Gaziosmanpaşa University, Tokat, Türkiye; ^11^Department of Physical Education and Sport, School of Physical Education and Sports, Harran University, Sanliurfa, Türkiye; ^12^Department of Sport Management, Faculty of Sports Sciences, Hitit University, Corum, Türkiye

**Keywords:** adolescent mental health, cognitive-behavioral therapy, physical activity interventions, ruminative thought patterns, subjective wellbeing

## Abstract

**Introduction:**

This study examined the associations among ruminative thinking style (RTS), subjective wellbeing (SWB), and cognitive-behavioral physical activity (CBPA) in a sample of 1,326 adolescents (aged 14–18) from sports high schools.

**Method:**

Using validated self-report measures, data were analyzed through bootstrapped mediation models.

**Results:**

RTS was negatively associated with SWB (β = −0.1792, *p* < 0.001), while CBPA showed a partial mediating effect in this relationship (indirect β = 0.0779, *p* < 0.001).

**Discussion:**

These results suggest that cognitive-behavioral orientations toward physical activity may buffer the negative impact of rumination on wellbeing. Consistent with cognitive-behavioral therapy (CBT) principles, CBPA could provide adolescents with self-regulatory strategies that support mental health. However, the cross-sectional and self-report design, as well as the focus on sports high school students, limit causal inference and generalizability. Future longitudinal and cross-cultural studies are needed to confirm these findings. Overall, the study provides preliminary evidence that CBPA-informed approaches may hold promise for promoting adolescents' mental wellbeing.

## 1 Introduction

The complexity of human thought processes, particularly the patterns that influence mental health, has become a central focus of modern psychology (Rogowska et al., 2020; Hayes and Hofmann, 2021; Wong et al., 2023). Ruminative thinking, defined as the repetitive focus on specific thoughts or events (Wen et al., 2024), is considered a key process in understanding how individuals experience and regulate emotions (Ratcliffe, 2023). From the perspective of cognitive theory, which underpins therapeutic approaches such as Cognitive-Behavioral Therapy (CBT), thoughts and beliefs shape emotions and behaviors (Prasko et al., 2024). Rumination exemplifies the maladaptive processing of such thought patterns. (Nolen-Hoeksema 1991), who conceptualized the construct, demonstrated its association with depression and other mental health problems, arguing that persistent focus on negative thoughts exacerbates psychological distress (Sürü, 2022). Research also shows that sport and physical activity may mitigate rumination and promote mental health (Michel-Kröhler et al., 2023; Parker et al., 2024). For instance, (Stubbs et al. 2016) found that attentional focus during exercise can interrupt ruminative cycles and facilitate a shift to more positive states (Morouço et al., 2024; Lewis, 2015). Similarly, (Coyle 2013) noted that improved cognitive processing skills through physical activity may help individuals reframe negative thoughts more adaptively.

Ruminative thinking has been shown to contribute to several mental health problems in adolescents, including persistent negative thoughts, depression, and anxiety (Espinosa et al., 2022). Elevated stress and anxiety during adolescence can further trigger rumination, which may disrupt social communication, strain peer relationships, and reduce self-confidence, thereby lowering overall mental wellbeing (Lin and Guo, 2024; Ghaffari et al., 2023). By contrast, CBT-based physical activity (CBPA) has been reported to reduce rumination by helping individuals disengage from negative thoughts (Miller-Mendes et al., 2023). In addition, such activity can enhance adolescents' wellbeing by fostering flow experiences through deep engagement in tasks (Pavlova et al., 2016; Carlsson et al., 2024). Consistent with this view, (Seligman 2011) emphasized that flow contributes to flourishing and overall wellbeing (Somjee, 2024).

CBT and physical activity, when combined in approaches targeting mental health, offer an effective strategy for psychological wellbeing (Geetanjali et al., 2023). CBT enables individuals to identify and modify maladaptive thought patterns, while physical activity reduces stress and enhances mood (Vela and Carroll, 2023). Exercise also facilitates affective regulation through mechanisms such as endorphin release (Mahindru et al., 2023; Hossain et al., 2024). Regular participation in physical activity improves mood and strengthens coping capacities. When integrated, CBT and physical activity allow individuals to manage emotions more effectively and support mental wellbeing (Martín-Rodríguez et al., 2024). Their combination is increasingly recognized as a comprehensive strategy for enhancing both psychological and physical health (Mohseni et al., 2022).

Building on these foundations, Cognitive-Behavioral Physical Activity (CBPA) has been conceptualized as more than participation in exercise. It incorporates cognitive–behavioral strategies—such as planning, goal setting, self-monitoring, and restructuring maladaptive thoughts—into physical activity routines (Eskiler et al., 2016). CBPA is therefore distinct from general physical activity, which emphasizes behavior alone, and from conventional CBT, which is applied in therapeutic contexts. Instead, CBPA operationalizes both behavioral engagement and cognitive regulation in everyday settings. In this study, CBPA was selected as a mediator because it theoretically reflects how adolescents' self-regulatory orientations toward activity may buffer the negative effects of rumination on wellbeing. Through such approaches, individuals can identify maladaptive thoughts, develop healthier coping strategies (Diachkova et al., 2024; Joubert et al., 2022), and move toward more positive mental states that enhance wellbeing (Azad and Marselle, 2025).

Given the lack of a systematic framework addressing how CBT and physical activity influence rumination and mental wellbeing (Hashemi Salehi and Aleyasin, 2024), this study seeks to contribute to this underexplored area. Specifically, it examines whether CBPA serves as a mediating mechanism in the relationship between ruminative thinking and subjective wellbeing among adolescents, focusing on a large sample of students from sports high schools.

## 2 Material and methods

### 2.1 Research group

The study was conducted with a sample of 1,326 adolescents aged 14–18 years from sports high schools. Recruitment was carried out with the aim of achieving approximate balance across both gender and age groups. Sports high schools were selected because students in these institutions generally engage in higher levels of structured physical activity and are therefore a relevant population for examining the role of CBPA. However, this context also limits the generalizability of the findings, as the results may not apply to adolescents in mainstream educational settings with lower activity levels. School rosters were screened to ensure proportional invitations by grade level and sex, and enrollment was monitored to maintain near-equal representation. The final sample comprised 670 males (50.5%) and 656 females (49.5%), with age groups distributed as follows: 14 years (*n* = 250, 18.9%), 15 years (*n* = 265, 20.0%), 16 years (*n* = 280, 21.1%), 17 years (*n* = 280, 21.1%), and 18 years (*n* = 251, 18.9%). The mean age of participants was 16.0 ± 1.39 years. The participants voluntarily participated in the study and signed an informed consent form. The sample size was determined using power analysis to ensure sufficient statistical power for mediation analyses (Fritz and MacKinnon, 2007). A priori power analysis (α = 0.05, 1–β = 0.80) assuming small-to-medium mediated effects (a=b≈0.14) indicated N≈…; our *N* = 1,326 exceeded this threshold. Data were collected using an online survey platform. The participants were informed about the purpose and procedures of the study, and their anonymity was guaranteed. The survey was completed in a single session lasting ~20 min. The rate of missing data was kept below 5%, and missing data checks were conducted to improve data quality. Ethical approval was obtained from the Tokat Gaziosmanpaşa University Ethics Commission (Approval No: 2025/544834, dated: April 3, 2025). Permissions were also obtained from school administrations at each site. All participants and their legal guardians provided written informed consent. A protocol was in place to identify and support at-risk participants (e.g., elevated distress scores); students were informed that they could withdraw at any time without penalty, and school counselors were available for referral when necessary. The study was conducted in accordance with the Declaration of Helsinki and local regulations.

### 2.2 Data collection tools

Three scales were used in this study: Ruminative Thinking Style (RTS), Cognitive Behavioral Physical Activity (CBPA), and Subjective WellBeing (SWB). The Ruminative Thinking Style Scale is a 20-item Likert-type scale for assessing individuals' tendencies toward ruminative thinking, and Cronbach's α value was found to be 0.934, indicating high internal consistency (Brinker and Dozois, 2009). The Turkish version of the scale was adapted by (Karatepe 2010). The Subjective WellBeing Scale was used to measure life satisfaction and emotional wellbeing, with a Cronbach's α value of 0.821. In this study, CBPA refers to cognitive–behavioral orientations toward engaging in physical activity (e.g., readiness, planning, self-regulatory strategies) rather than objective activity volume (e.g., duration or intensity), and it was assessed with the Cognitive Behavioral Physical Activity Scale (Eskiler et al., 2016; α = 0.801). These values indicate that the scales meet the reliability standards accepted in the social sciences (Nunnally and Bernstein, 1994). Beyond reliability, prior studies demonstrated the construct validity of all scales used in this study. The Ruminative Thinking Style Scale has shown strong convergent validity with depression and anxiety measures (Brinker and Dozois, 2009; Karatepe, 2010). The Subjective WellBeing Scale demonstrated factorial validity in both the original (Diener et al., 2010) and Turkish adaptation studies (Fidan and ve Usta, 2013). The Cognitive Behavioral Physical Activity Scale (Eskiler et al., 2016) showed acceptable factorial validity and criterion-related validity with physical activity engagement and motivation. These psychometric properties support the validity of the measures in adolescent samples.

### 2.3 Data analysis

Mediation models were employed to examine the relationships among Ruminative Thinking Style (RTS), Cognitive-Behavioral Physical Activity (CBPA), and Subjective WellBeing (SWB). Analyses were conducted using R software version 4.1 (R Core Team, 2021) and the jAMM module on the jamovi platform (Gallucci, 2020; The Jamovi Project, 2022). Two models were tested: (1) Model M1 assessed the prediction of CBPA by RTS, and (2) Model M2 assessed the prediction of SWB by both CBPA and RTS. Mediation effects (IE1: RTS ⇒ CBPA ⇒ SWB) were estimated with 5,000 bootstrap replications, and 95% confidence intervals were reported. Standardized regression coefficients (β) were presented as effect sizes, with significance evaluated at *p* < 0.05 (Rosseel, 2012). The internal consistency of all scales was assessed with Cronbach's α using the psych package (Revelle, 2019).

Prior to mediation analyses, statistical assumptions were verified. Normality of residuals and multicollinearity were inspected; variance inflation factors (VIF) were < 2, indicating no collinearity concerns. Outliers and influential cases were assessed with leverage values and Cook's distance, and none exceeded recommended thresholds. As a robustness check, demographic variables (age and gender) were added as covariates in supplementary models. The mediation results remained stable across these tests, suggesting that the observed associations are not attributable to statistical artifacts or demographic confounders. These procedures increase confidence in the credibility of the effect sizes and the validity of the mediation model.

## 3 Findings

For transparency, all effects are reported with standardized β, standard errors (SE), z values, *p-*values, and 95% percentile bootstrap confidence intervals. Reporting was harmonized across all tables and figures. The analytic sample (*N* = 1,326) was approximately balanced by gender [Male = 670 (50.5%); Female = 656 (49.5%)] and age [14: 250 (18.9%), 15: 265 (20.0%), 16: 280 (21.1%), 17: 280 (21.1%), 18: 251 (18.9%)]. The mean age was 16.0 ± 1.39 years. These distributions are consistent with our stratified recruitment plan across grade levels and schools.

The Cronbach's α values indicate high internal consistency for all measures. The Ruminative Thinking Style Scale demonstrated excellent reliability (α = 0.934; Tabachnick and Fidell, 2013). The Subjective WellBeing (α = 0.821) and Cognitive-Behavioral Physical Activity (α = 0.801) scales also exceeded the 0.80 threshold, reflecting good reliability in the context of social sciences (Field, 2024). The observed means and standard deviations suggested relatively homogeneous distributions, with scale scores falling in the moderate range. Overall, the instruments used in this study provide evidence of adequate validity and reliability (Table 1).

**Table 1:** Descriptive statistics and internal consistency (Cronbach's α) of the study scales.

**Scales**	**Standard deviation**	**Mean**	**Cronbach's α**
Ruminative thought style	4.15	1.37	0.934
Subjective wellbeing	4.72	1.29	0.821
Cognitive behavioral physical activity	3.40	0.62	0.801

In this study, the relationships between Ruminative Thinking Style (RTS), Cognitive-Behavioral Physical Activity (CBPA), and Subjective WellBeing (SWB) were examined using mediation models. In the first model (M1), CBPA was significantly predicted by RTS, suggesting that individuals with higher levels of rumination may show reduced cognitive-behavioral engagement in physical activity. In the full model (M2), both RTS and CBPA were included as predictors of SWB. The results indicated that CBPA was a significant positive predictor of SWB, while RTS exerted both direct negative and indirect effects. Specifically, the indirect effect (IE1) analysis indicated that RTS was associated with SWB partly via CBPA, consistent with a partial mediating role in this cross-sectional model. These findings imply that cognitive-behavioral dispositions toward physical activity may buffer the adverse impact of rumination on wellbeing. The analyses were based on a large sample of 1,326 adolescents, which increased statistical power; however, the generalizability of the results remains limited to the sports high school context (Table 2).

**Table 2:** Mediation model specifications and sample information.

**Models info**		
Mediators models	M1	CBPA ~ RTS
Full model	M2	SWB ~ CBPA + RTS
Indirect effects	IE1	RTS ⇒ CBPA ⇒ SWB
Sample size	N	1,326

CBPA, Cognitive Behavioral Physical Activity; RTS, Ruminative Thinking Style; SWB, Subjective WellBeing.

**Figure 1:**
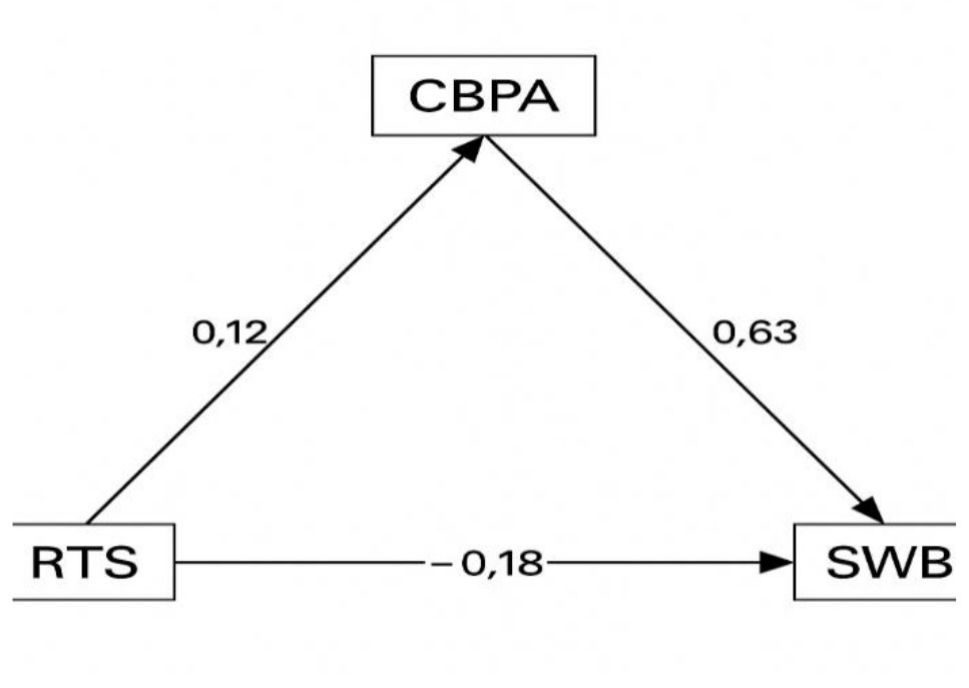
Statistical path diagram.

The structural model suggested that cognitive-behavioral dispositions toward physical activity were associated with adolescents' wellbeing, with CBPA partly mediating the association between rumination and wellbeing. In addition to its direct negative effect on wellbeing, ruminative thinking style also exerted an indirect positive effect through CBPA. These findings are compatible with the potential utility of CBPA-informed approaches for adolescents with higher rumination, to be tested in future longitudinal or experimental studies, as shown in Figure 1.

*When* *Table 3* *is analyzed, the following results are obtained:*

**Table 3:** Mediation analysis results: effects of RTS on SWB through CBPA.

**Type**	**Effect**	**Estimate**	**SE**	**95% C.I**.		**β**	**z**	**p**
				**Lower**	**Upper**			
Indirect	RTS ⇒ CBPA ⇒ SWB	0.0294	0.00739	0.0160	0.0446	0.0779	3.98	< 0.001
Component	RTS ⇒ CBPA	0.0402	0.00962	0.0224	0.0589	0.1243	4.18	< 0.001
	CBPA ⇒ SWB	0.7319	0.03023	0.6735	0.7943	0.6269	24.21	< 0.001
Direct	RTS ⇒ SWB	−0.0676	0.00887	−0.0858	−0.0511	−0.1792	−7.62	< 0.001
Total	RTS ⇒ SWB	−0.0382	0.01176	−0.0616	−0.0144	−0.1013	−3.25	< 0.001

CBPA, Cognitive Behavioral Physical Activity; RTS, Ruminative Thinking Style; SWB, Subjective WellBeing. Standardized β coefficients are reported. Confidence intervals were estimated using 5,000 bootstrap resamples (percentile method). Effects were considered statistically significant at p < 0.05.

Indirect Effect: RTS ⇒ CBPA ⇒ SWB

The unstandardized indirect effect was 0.0294 [SE = 0.00739, 95% CI (0.0160, 0.0446)], corresponding to a standardized effect size of β = 0.0779 (*z* = 3.98, *p* < 0.001). This finding suggests that CBPA plays a partial mediating role in the relationship between ruminative thinking styles and subjective wellbeing. This effect comprises two components.

1. RTS ⇒ CBPA the path effect coefficient β = 0.1243, *z* = 4.18, *p* < 0.001 indicates that CBPA is significantly predicted by the RTS.

2. For the CBPA ⇒ SWB path, a large standardized association was observed (β = 0.6269, *z* = 24.21, *p* < 0.001), indicating that CBPA is a robust positive predictor of SWB.

Direct Effect: RTS ⇒ SWB

The direct effect of RTS on SWB was negative and statistically significant (β = −0.1792, *z* = −7.62, *p* < 0.001). This finding shows that as individuals' ruminative thinking styles increase, their subjective wellbeing levels decrease. In other words, rumination tendency directly affects wellbeing negatively.

Total Effect: RTS ⇒ SWB

The total effect of RTS on SWB was β = −0.1013 (*p* < 0.001), which corresponds to the sum of the direct and indirect effects. Since the total effect has a lower absolute value than the direct effect, the negative effect of ruminative thinking is somewhat attenuated by the positive indirect effect of CBPA. Cognitive behavioral physical activity plays a statistically significant partial mediating role in the relationship between individuals' ruminative thinking styles and their subjective wellbeing levels. In this context, increasing cognitive and behavioral orientations toward physical activity may reduce the negative effects of ruminative thinking on subjective wellbeing. Developing CBPA, especially in intervention programs or psychoeducation-based practices, may be an effective strategy to regulate mental processes and support wellbeing. Robustness checks confirmed that the mediation results remained consistent after including demographic covariates and after excluding potential outliers. Effect sizes and significance levels did not change materially.

## 4 Discussion

This study examined the relationships among ruminative thinking style (RTS), subjective wellbeing (SWB), and cognitive-behavioral physical activity (CBPA) in sports high school students aged 14–18 years, with a focus on the mediating role of CBPA. The findings indicated that CBPA functioned as a statistically significant partial mediator in this cross-sectional model, suggesting an association whereby CBPA may attenuate the link between RTS and SWB (β = 0.0779, *p* < 0.001). While RTS directly predicted lower levels of SWB (β = −0.1792, *p* < 0.001), it also showed an indirect positive effect through CBPA. These results are consistent with previous studies and support the notion that physical activity may help reduce the negative effects of rumination and promote mental wellbeing (Mahindru et al., 2023; Martín-Rodríguez et al., 2024).

Rumination has been linked to mental health problems such as depression, anxiety, and diminished self-confidence in adolescents (Espinosa et al., 2022; Ghaffari et al., 2023). Consistent with this evidence, the present study demonstrated a direct negative effect of RTS on SWB, indicating that higher levels of rumination are associated with lower levels of wellbeing. Interestingly, RTS also positively predicted CBPA, contrary to the expectation that rumination would reduce cognitive-behavioral orientations toward activity. One possible explanation is that sports high school students, despite experiencing rumination, may channel these repetitive thoughts into structured engagement with physical activity as a coping mechanism. Alternatively, the CBPA construct emphasizes planning and cognitive monitoring, processes that may be elevated in ruminative individuals. Future studies should clarify whether this positive association generalizes beyond the sports school context. At the same time, the mediating role of CBPA suggests that it may buffer these adverse effects to some extent. Prior research has shown that physical activity can help individuals disengage from negative thoughts and shift toward more positive mental states, partly through mechanisms such as endorphin release (Hossain et al., 2024). In this context, the strong positive association observed between CBPA and SWB (β = 0.6269, *p* < 0.001) supports the view that physical activity contributes not only to physical health but also to psychological wellbeing. Nevertheless, the indirect effect identified in this study was modest, and thus these interpretations should be considered preliminary (Mohseni et al., 2022). Given the cross-sectional and self-report design, the proposed mechanisms remain tentative and require confirmation in future longitudinal and experimental research.

Theoretically, CBPA may serve as a mediator because it incorporates both cognitive and behavioral processes that are central to mental wellbeing. From a CBT perspective, CBPA reflects self-regulatory strategies such as planning, monitoring, and reframing negative thoughts, which may reduce the intensity of ruminative thinking. At the same time, engagement in physical activity introduces physiological and experiential mechanisms—such as endorphin release and immersion in flow-like states—that are associated with enhanced mood and wellbeing. The combination of these elements provides a dual pathway: cognitive restructuring helps adolescents reinterpret negative experiences, while physical activity provides immediate affective and physiological benefits. This integration may explain why adolescents with stronger cognitive–behavioral orientations toward physical activity experience attenuated associations between rumination and lower wellbeing. Therefore, CBPA is not only statistically but also theoretically meaningful as a mediator, linking ruminative thought patterns with subjective wellbeing.

The findings of this study are consistent with the view that CBT-based physical activities are associated with lower levels of rumination and may represent a promising approach to support adolescents' mental health. While CBT enables individuals to identify and restructure maladaptive thought patterns, physical activity complements this process by enhancing stress-coping capacity (Hashemi Salehi and Aleyasin, 2024; Diachkova et al., 2024). In this context, CBPA may help foster self-esteem and a sense of accomplishment by reducing ruminative thinking, thereby supporting mental wellbeing. These findings also align with Seligman's (2011) flow theory, which proposes that full engagement in activity can enhance wellbeing by facilitating deeper psychological involvement (Somjee, 2024).

It has been reported that sports can facilitate a shift to a more positive mental state by temporarily interrupting ruminative thinking (Stubbs et al., 2016). In this study, however, CBPA was shown not only to provide temporary relief but also to demonstrate a consistent mediating role in the relationship between rumination and wellbeing. This pattern motivates further evaluation of CBPA-informed school programs in longitudinal or randomized designs, rather than supporting immediate implementation. Such research could clarify whether integrating cognitive-behavioral strategies into physical activity programs may help adolescents manage rumination and improve wellbeing (Lin and Guo, 2024). For example, integrating physical activity programs in sports high schools with cognitive–behavioral components may represent a feasible and promising approach to supporting adolescents' mental health.

A key contribution of this study is that it systematically examines the mediating role of CBPA in a large sample of sports high school adolescents (*N* = 1,326). In the literature, there are limited studies focusing on the mediating effects of CBPA in this age group (Hashemi Salehi and Aleyasin, 2024). This study adds to the literature by examining the role of CBPA in adolescents' mental wellbeing within a sports high school context, an underrepresented population, and by applying mediation analysis in a large sample. Moreover, the large sample of 1,326 participants increases the statistical power and generalizability of the findings.

This study is not without limitations. First, the cross-sectional design precludes firm causal inferences, and future research should examine the long-term effects of CBPA using longitudinal approaches. Second, the study focused exclusively on sports high school students; therefore, the generalizability of the findings is limited to similar educational contexts. Third, CBPA was assessed solely through self-report measures; future studies could incorporate objective indicators (e.g., physiological markers) to strengthen the validity of the results.

This study showed that CBPA functions as a statistically significant, though modest, mediator in the relationship between ruminative thinking and mental wellbeing. These findings provide preliminary evidence that CBPA-based interventions may help support adolescent mental health. Integrated CBT and physical activity programs in sports high schools may represent a promising approach, although the present results indicate attenuation of negative effects of rumination rather than causal reduction. Future research should build on these findings by examining CBPA across different age groups and educational contexts.

The results of this study support the view that ruminative thinking negatively affects mental wellbeing during adolescence and is associated with psychological problems such as depression and anxiety (Espinosa et al., 2022). At the same time, the mediating role of CBPA suggests that it may offer a potential mechanism for mitigating the detrimental effects of rumination on wellbeing. This interpretation is consistent with prior research indicating that physical activity can help individuals break negative thought cycles, foster positive mental states, and support emotional wellbeing through physiological processes such as endorphin release (Hossain et al., 2024). The strong positive effect of CBPA on SWB (β = 0.6269, *p* < 0.001) further suggests that physical activity may play an important role in both physical and psychological health (Mohseni et al., 2022). These findings align with the idea that CBT-based physical activities may reduce ruminative thinking and foster healthier cognitive structures, thereby enhancing adolescents' ability to cope with stress (Hashemi Salehi and Aleyasin, 2024). They are also compatible with Seligman's (2011) flow theory, which posits that full engagement in physical activities can enhance adolescents' mental wellbeing (Somjee, 2024).

From a practical perspective, the findings suggest that CBPA-oriented activities may be systematically integrated into school-based physical education or extracurricular sports programs. For instance, short and structured sessions (e.g., 20–30 min, 2–3 times per week) that combine physical exercise with cognitive–behavioral strategies such as goal setting, self-monitoring, and guided reflection could be feasible in sports high school settings. Teachers and coaches can be trained to embed CBT principles into routine practice by encouraging students to identify negative thoughts, reframe them, and set achievable performance goals during activity. Such interventions may be adapted in intensity depending on students' age and fitness levels, and their feasibility is enhanced by utilizing existing school resources and personnel. Overall, CBPA-based programs may hold promise as feasible and scalable approaches to promote adolescent mental wellbeing, provided they are carefully structured and systematically monitored. Given the cross-sectional, self-report design, reverse causation and residual confounding cannot be ruled out; thus, the mediation estimates should be interpreted as statistical rather than causal mediation.

## 5 Conclusion

In conclusion, this study provides evidence of statistical associations consistent with a partial mediating role of CBPA in the relationship between ruminative thinking and subjective wellbeing in the relationship between ruminative thinking and subjective wellbeing among adolescents in sports high schools. The findings highlight the potential value of CBPA-oriented approaches for supporting mental health in this population. However, given the cross-sectional and self-report design, causal inferences cannot be drawn, and the observed mechanisms should be regarded as hypothetical. Future longitudinal and experimental research is needed to establish temporal ordering, test underlying mechanisms, and examine generalizability across more diverse adolescent groups. The dissemination of CBT-integrated physical activity programs may therefore be considered a promising, yet preliminary, strategy to enhance adolescents' mental wellbeing by reducing their tendency toward ruminative thinking.

## 6 Limitations

This study was conducted with a large sample (*N* = 1,326), which increased statistical power and reliability. However, its cross-sectional design precludes causal inference. Although the constructs were statistically distinct, conceptual overlap may limit discriminant validity. While age and gender were controlled for, other potential confounders (e.g., socioeconomic status, prior mental health) were not assessed. The relatively strong effect sizes may partly reflect common-method variance due to the self-report format; future studies should apply statistical checks (e.g., Harman's single-factor test) and incorporate multi-method approaches to reduce this risk. Objective measures, such as accelerometers for physical activity and behavioral tasks for rumination, would also strengthen validity. Finally, participants were recruited exclusively from sports high schools, a subgroup characterized by high physical activity, which restricts generalizability to broader adolescent populations. Future research should examine more diverse educational and cultural contexts.

## 7 Recommendations

These findings suggest a potential value in designing and implementing CBPA-oriented intervention programs to support adolescents' mental health. However, given the cross-sectional design, causal inferences cannot be drawn, and longitudinal studies are required to establish temporal ordering. The dissemination of CBT-integrated physical activity programs in sports high schools may, therefore, be considered a promising but preliminary strategy to enhance adolescents' mental wellbeing by reducing their tendency toward ruminative thinking.

The manuscript addresses a relevant and timely topic with a large adolescent sample, which is a notable strength. Nevertheless, revisions are needed in both the Methods and Discussion sections to enhance clarity, ensure more cautious interpretation of findings, and strengthen theoretical integration. Addressing these issues will improve the manuscript's transparency, methodological rigor, and overall credibility, thereby increasing its potential contribution to the literature on adolescent mental health and cognitive-behavioral approaches to physical activity.

## Data Availability

The original contributions presented in the study are included in the article/supplementary material, further inquiries can be directed to the corresponding author.
